# Case Report: Pemphigoid Nodularis—Five Patients With Many Years of Follow-Up and Review of the Literature

**DOI:** 10.3389/fimmu.2022.885023

**Published:** 2022-04-12

**Authors:** Konrad Szymanski, Alicja Adaszewska, Beata Jakubowska, Cezary Kowalewski, Ewelina Pietrzyk, Katarzyna Wozniak

**Affiliations:** Department of Dermatology, Immunodermatology and Venereology, Medical University of Warsaw, Warsaw, Poland

**Keywords:** pemphigoid nodularis, nodular mucous membrane pemphigoid, clobetasol propionate, antidepressants, treatment

## Abstract

Pemphigoid nodularis is a rare form of pemphigoid that joins the clinical picture of prurigo nodularis and the immunological features of bullous pemphigoid, which is therapeutically challenging. Here, we analyze five female patients with a long-lasting course of nodular pemphigoid in terms of clinical and immunological characteristics and therapy. All the patients fulfilled clinical and immunological criteria of nodular pemphigoid. We applied numerous techniques allowing the proper diagnosis: direct and indirect immunofluorescence, salt split skin, ELISA, BIOCHIP, and fluorescence overlay antigen mapping using laser scanning confocal microscopy. Our study showed that 4 of 5 patients fulfilled the clinical and immunological criteria of nodular bullous pemphigoid. Two out of 4 patients presented exclusively nodular lesions; in the other two patients, blisters and erythematous lesions preceded prurigo-like lesions by a few years. The remaining patient had clinical and immunological criteria of nodular mucous membrane pemphigoid, presenting oral erosions, scarring conjunctivitis, and numerous disseminated nodules on the skin. All the patients were treated with multiple medicines; however, it was observed that the use of clobetasol propionate on the entire body plus antidepressants best controlled the disease. Pemphigoid nodularis mainly occurs in elderly women. In cases with coexisting psychological problems, antidepressants should be considered as an important complementary therapy to the basic one with clobetasol propionate.

## Introduction

Bullous pemphigoid (BP) is the most common autoimmune subepidermal blistering disease equally affecting men and women over 60 years of age (1). The disease is characterized by the development of autoantibodies against the NC16a domain of BP180, an antigen of the basement membrane zone (BMZ) (1). This leads to the characteristic clinical picture consisting of erythematous and edematous lesions as well as tense blisters localized on the trunk and extremities (2). However, it is widely accepted that BP may present unusual clinical features, like dyshidrotic, localized erythroderma (2). Pemphigoid nodularis (PN) is another rare form of pemphigoid that occurs more frequently in older women (3–5). This variant of BP is extremely challenging in terms of diagnostics and therapy since it may be clinically indistinguishable from prurigo nodularis. It is noteworthy that the etiology of prurigo nodularis is not clear, but neuroimmune alterations play a role. It is also considered that prurigo nodularis is associated with other dermatological conditions (atopic dermatitis, and xerosis), systemic disorders (renal failure), as well as medications. It is also stressed that psychiatric disorders accompanied by pruritus significantly predispose to prurigo nodularis (6). However, the main difference between PN and prurigo nodularis is the lack of characteristic immunological findings in patient’s skin and serum.

Here, we present five female patients with PN presenting different immunological characteristics, course, and therapy.

## Materials and Methods

### Patients

The study included 5 patients, all women (mean age, 74 years; range 62–85) (detailed characteristics are depicted in **Table 1**), who presented clinical and immunological criteria of PN. The clinical criterion was the presence of long-lasting disseminated nodules accompanied by pruritus. The immunological criterion was positive direct immunofluorescence (DIF) with the presence of linear IgG and/or C3 along the BMZ and indirect immunofluorescence (IIF) revealing circulating anti-BMZ antibodies (**Table 2**).

**Table 1:** Clinical and therapeutic characteristics of patients included in the study.

No	Sex/Age	History of nodular pemphigoid	Comorbidities	Type of skin lesions and location	Treatment	
					Unsuccessful	Successful
P1	F/85	12-year duration with numerous relapses	HT	Exclusively nodules on the trunk and limbs during entire course + severe pruritus	Prednisone, tetracycline, methotrexate	Clobetasol propionate, mirtazapine + duloxetine
P2	F/62	9-year duration The longest remission lasted several months	HT, COPD, anxiety disorder	Initially disseminated blisters and urticarial lesions, then only nodules + severe pruritus	Prednisone, methyloprednisone, tetracycline, methotrexate, oral antihistaminics	Clobetasol propionate cream on the entire body
P3	F/80	1.5 years before therapy 7-month therapy led to the complete remission with no relapse	HT	Exclusively nodules on the trunk and limbs during entire course + severe pruritus		Exclusively clobetasol propionate cream on the entire body
P4	F/67	9-year duration Conjunctiva and oral mucosa in stable state for several years Nodules on the skin occasionally appear	T2DM, HT, asthma, obesity	Extensive oral erosions, scarring conjunctivitis, nodules on the trunk and limbs + severe pruritus	Clobetasol propionate lesionally, tacrolimus, antihistaminics, prednisone, escitalopram, paroxetine	Dapsone clomipramine + quetiapine
P5	F/77	10-year duration With remissions lasting a few months	Obesity, HT, CHD, HF, T2DM, PBC, sleep apnea, depression	Initially disseminated urticarial lesions and few blisters, then only numerous nodules + severe pruritus	Antihistaminics, prednisone, methyloprednisone, tetracycline, methotrexate, dapsone	Clobetasol propionate cream on the entire body escitalopram

CHD, coronary heart disease; COPD, chronic obstructive pulmonary disease, HF, heart failure; HT, hypertension; PBC, primary biliary cirrhosis; T2DM, type 2 diabetes.

**Table 2:** Immunological characteristics of patients included in the study.

No.	DIF	Serum studies	FOAM-LSCM
P1	Skin: exclusively linear C3 along the BMZ	**BIOCHIP:** positive reaction of circulating IgG on the roof of the blister of salt split skin and with BP230 **ELISA:** negative	ND
P2	Skin: linear deposits of IgG and C3 along the BMZ	**IIF on SSS:** circulating IgG on the roof of the blister **Both BIOCHIP and ELISA:** reaction of circulating IgG antibodies with NC16a domain of BP180	ND
P3	Skin: linear deposits of IgG and C3 along the BMZ	**IIF on SSS:** circulating IgG on the roof of the blister **Both BIOCHIP and ELISA** were negative	ND
P4	Skin: linear deposits of IgG and C3 along the BMZ Oral mucosa: deposits of IgG along the BMZ Conjunctiva: deposits of IgG along the BMZ	**IIF on SSS:** circulating IgG on the roof of the blister **ELISA:** reaction of circulating IgG antibodies with BP230	In both skin and oral mucosa—IgG deposits located below laminin 332 and above collagen type IV, typically for MMP
P5	Skin: linear deposits of IgG and C3 along the BMZ	**IIF on SSS:** circulating IgG on the roof of the blister **Both BIOCHIP and ELISA:** reaction of circulating IgG antibodies with NC16a BP180 and BP230	ND

BMZ, basement membrane zone; ELISA, enzyme-linked immunosorbent assay; FOAM-LSCM, fluorescence overlay antigen mapping using laser scanning confocal microscopy; IIF, indirect immunofluorescence; MMP, mucous membrane pemphigoid, ND, not done; SS, salt split.

The study was approved by the Bioethical Committee of the Medical University of Warsaw. Informed and written consent form was obtained from the participants.

### Methods

#### Direct Immunofluorescence

Direct immunofluorescence was performed on patient’s normal-appearing tissue according to a previously described method (7).

#### Indirect Immunofluorescence and on Salt Split Skin

Patients’ sera were studied by routine indirect immunofluorescence method (IIF) and on the salt split skin substrate (SS-IIF) according to the method described previously (8).

#### Enzyme-Linked Immunosorbent Assay for BP180 and BP230

Anti-BP180 and anti-BP230 antibodies were studied with commercially available ELISA kits (Euroimmun, Lubeck, Germany) that employ the extracellular domain of recombinant proteins BP180 NC16a and BP230. Assays were carried out in accordance with the manufacturer’s instructions. The results were measured by an ELISA plate reader (KHB-ST 360). The standard recommended cutoff values were 20 RU/ml for both BP-180 NC16a and BP-230 (9).

#### BIOCHIP Mosaics Technique

In our study, we used the BIOCHIP Dermatology Mosaic 7 (Euroimmun, Lubeck, Germany), which consists of (i) monkey esophagus (BIOCHIP-ME), (ii) salt split skin (BICHIP-SSS), (iii) recombinant BP180 NC16a, (iv) HEK293 cells transfected with BP 230, (v) HEK293 cells transfected with Dsg1, and (vi) HEK293 cells transfected with Dsg3. The assay was carried out in accordance with the manufacturer’s instructions (9).

#### Fluorescence Overlay Antigen Mapping Using Laser Scanning Confocal Microscopy

The FOAM-LSCM technique was performed according to the method described previously (10). The tissue sections were incubated with mouse monoclonal antibodies directed against BMZ markers: antibody to laminin-332 (Chemicon, MAB 1949) directed against epitope of laminin-332 located in the lower lamida lucida was used as a marker of the lamina lucida–lamina densa border, whereas antibody to type IV collagen (Sigma, clone col-94) served as a marker of the lamina densa. In the second stage, the sections were treated with a conjugate mix: Cy5-labeled anti-mouse IgG antibody giving a red stain (Chemicon, AP160S) and anti-human IgG antibodies conjugated with FITC giving a green stain (Cappel, alfa chain). A confocal microscope Radiance 2000 with an appropriate means of laser lines for Cy5 and FITC (637 and 488 nm) and appropriate filters (a long-pass filter of 660 nm for Cy5 and a bandpass filter of 500–560 nm for FITC) was used to avoid cross-reactivity of reagents (11).

## Case Reports and Results

Characteristics of the patients are depicted in **Tables 1**, **2**.

### Case 1

**Figure 1:**
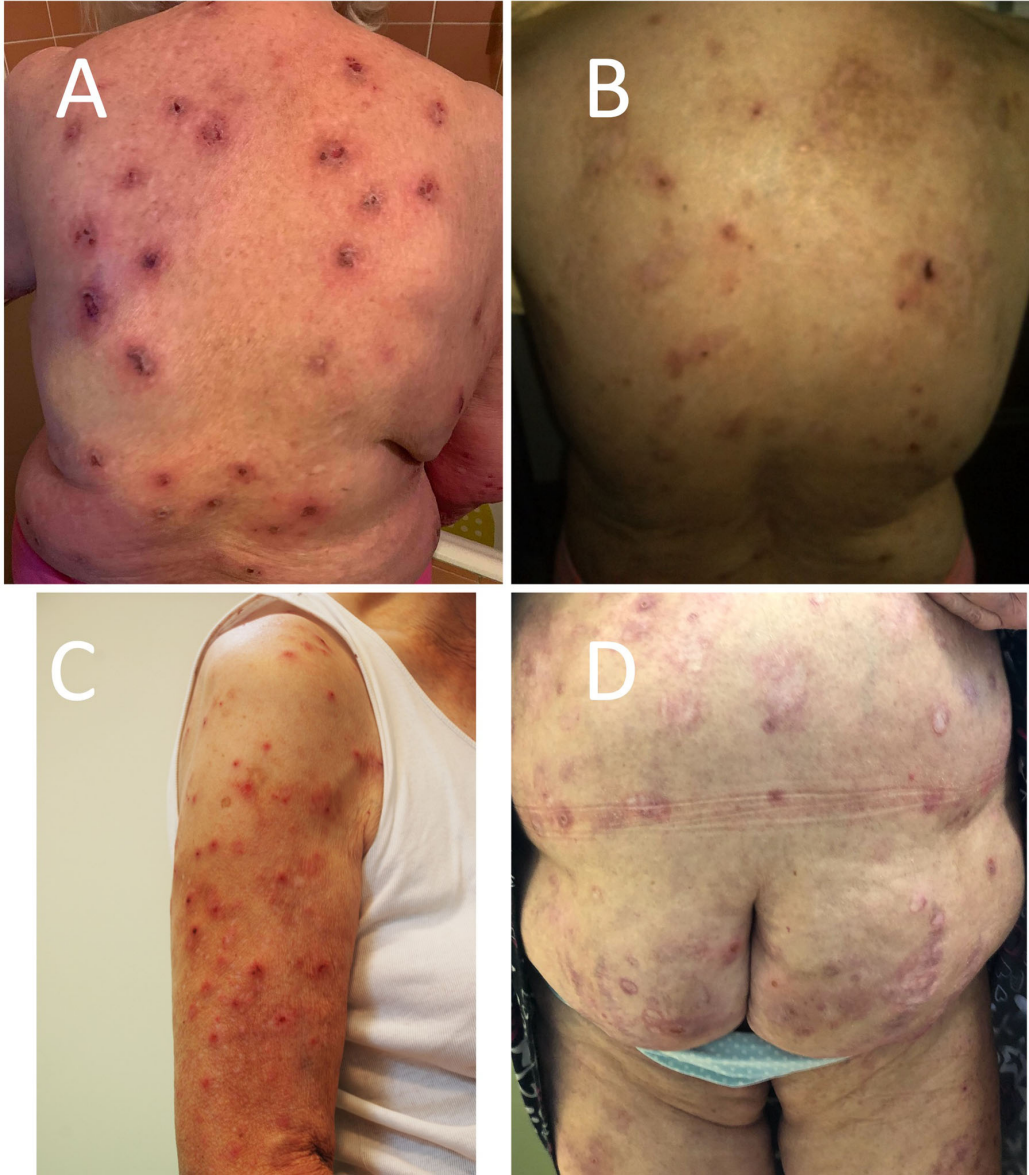
Clinical manifestation of pemphigoid nodularis in the studied patients—disseminated nodules, excoriations, hyperpigmentation, and hypopigmentation in **(A)** patient 1 (P1), **(B)** patient 2 (P2), **(C)** patient 3 (P3), and **(D)** patient 5 (P5).

The first case involves an 85-year-old woman with hypertension and with a 12-year history of BP. The onset of the disease was characterized by disseminated nodules and severe pruritus; thus, she was considered as prurigo nodularis. The diagnosis of BP was established 1 year later. During the entire course, the patient presented exclusively numerous nodules, excoriations, and subsequently postinflammatory hyperpigmentation with any blisters (**Figure 1A**). Initially, she was treated with oral prednisone 40 mg/day, tetracycline 2×500 mg/day, and methotrexate 15 mg/week with no significant improvement. Repeated DIF of perilesional skin consistently revealed linear deposition of C3 only along the BMZ. She never presented IgG deposits at the BMZ. IIF repeated a few times was negative. The BIOCHIP examination showed positive reaction of circulating IgG on salt split skin and with BP230 BMZ antigen. ELISA test for BP230 and BP180 was negative. Combined therapy of topical clobetasol 25 g/day (12) and mirtazapine 15–30 mg/day resulted in very good clinical response along with itching reduction. Unfortunately, the patient was not cooperating and unsystematic with the treatment, causing recurrences of nodules. Recently, she was diagnosed with depression and started duloxetine. Adding prednisone 40 mg/day and clobetasol propionate topically led to the significant reduction of itch and disappearance of nodules. The dose of prednisone has been gradually tapered to 5 mg used every other day. The patient has been in remission for 6 months.

### Case 2

A 62-year-old woman with hypertension, chronic obstructive pulmonary disease, and anxiety disorder was referred to our clinic in 2009 year with a 3-year history of BP presenting erythemas and large blisters on the trunk and extremities with severe pruritus. For the next 6 years, we observed alternating remissions and relapses. During the last 2 years of observation in our clinic, the patient presented only nodules without any blister formation (**Figure 1B**).

Immunological tests were consistent with the diagnosis of BP, i.e., DIF showed linear deposits of IgG and C3 at the BMZ. IIF disclosed circulating IgG anti-BMZ antibodies. Both BIOCHIP and ELISA disclosed reaction of circulating IgG antibodies with the NC16a domain of BP180. The patient was treated with oral prednisone, methyloprednisone, tetracycline, methotrexate, and oral antihistaminic without long-term remission achievement. Finally, topical clobetasol propionate in cream (12) applied on the entire body led to very quick (in 3 weeks) disappearance of nodules. The patient was observed for the next several months without relapse and then she failed to continue the follow-up.

### Case 3

An 80-year-old patient with hypertension was referred to our clinic in May 2015 with a 1.5-year history of pruritic papules that subsequently transformed into nodules involving her trunk and limbs (**Figure 1C**). There has never been any evidence of blister formation or erythema. DIF revealed linear deposits of IgG and C3 along the BMZ. On IIF, circulating IgG anti-BMZ antibodies were detected labeling the roof of the blister on SS-IIF. The BIOCHIP and ELISA were negative in terms of target antigens. The patient was treated with topical clobetasol with an initial dose of 25 g of cream per day on the entire body resulting in complete remission of skin lesions and itching in the first 2 weeks. Then, the cream application was continued with progressive dose reduction according to the original description (12) for a few months without recurrence of skin lesions. She has been in clinical remission for 6 years.

### Case 4

**Figure 2:**
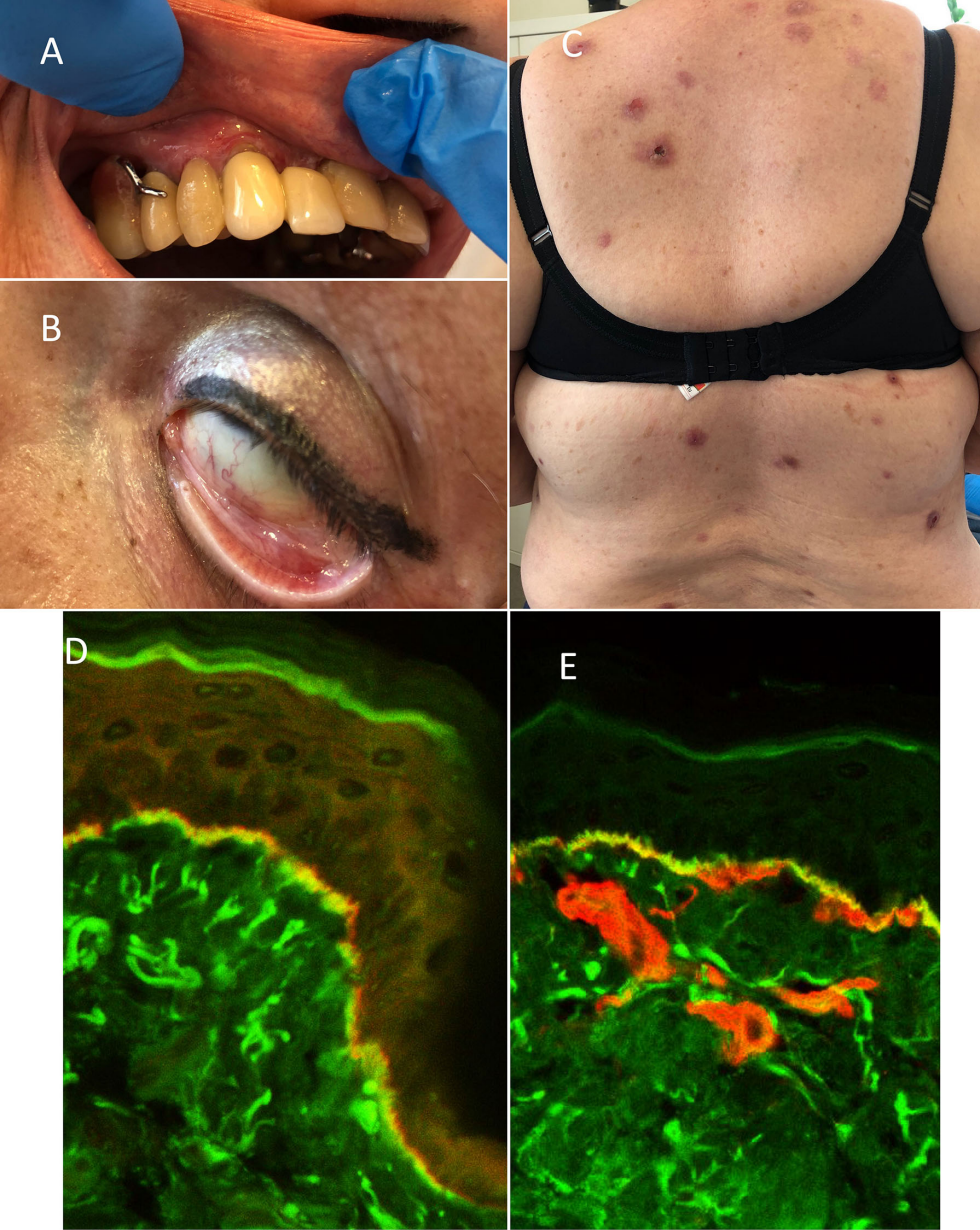
Clinical and immunological features of patient 4 (P4): **(A)** gingivitis, **(B)** scarring conjunctivitis of the left eye, and **(C)** disseminated nodules located on the trunk. FOAM-LSCM of patient’s oral mucosa: **(D)** linear IgG deposits (green color) localized below laminin-332 (red color) and **(E)** above collagen type IV (red color) at the BMZ.

A 67-year-old woman with type 2 diabetes, hypertension, asthma, and obesity was referred to our outpatient clinic 7 years ago with a 2-year history of extensive oral erosions on gingiva, palates, and buccal mucosa and single erosions on the trunk (**Figures 2A–C**). The DIF performed on oral mucosa and skin revealed IgG and IgA deposits along the BMZ. Overlay antigen mapping using laser scanning confocal microscopy disclosed IgG deposits below laminin 332 and above collagen type IV, typically for mucous membrane pemphigoid (MMP) (**Figures 2D, E**). Topical treatment with clobetasol propionate and tacrolimus for a few weeks was not effective; therefore, prednisone 40 mg/day and dapsone 100 mg/day were launched, leading to the remission lasting 2 years. After self-discontinuation of therapy, she relapsed oral erosions and developed scarring conjunctivitis in the left eye. DIF performed on patient’s conjunctiva showed *in vivo* bound IgG along the BMZ confirming MMP. Dapsone in combination with prednisone 0.2 mg/kg b.m. resulted in complete remission of MMP. However, during therapy, skin itching, initially periodic, then later became permanent as well as nodules on the trunk and extremities were reported by the patient. In repeated DIF from skin biopsy, linear deposits of IgG and IgA at the BMZ were detected, confirming the diagnosis of nodular MMP. ELISA revealed the presence of IgG antibodies against BP230 antigen only. The dapsone therapy was enriched in clobetasol propionate in cream lesionally as well as antidepressants such as escitalopram and paroxetine, which caused slight reduction of itch. Eventually, launching clomipramine with quetiapine led to the dramatic reduction of itch and prevented the formation of new nodules.

### Case 5

A 77-year-old woman with numerous comorbidities (obesity, hypertension, ischemic heart disease, heart failure, type 2 diabetes, primary biliary cirrhosis, sleep apnea, and depression) was diagnosed with BP 10 years ago. The diagnosis was based on DIF (IgG and C3 deposits along the BMZ), IIF on salt split skin (circulating anti-BMZ IgG antibodies reacted with the roof of an artificial blister), and ELISA (positive reaction of circulating IgG antibodies with the NC16a domain of BP180 antigen). Initially, she presented classical features of BP (blisters and erythematous lesions accompanied by pruritus). At that time, she was treated with numerous medicines (prednisone, dapsone, and methotrexate) that had to be discontinued due to side effects. Then, the patient was treated with topical clobetasol propionate with an initial dose of 40 g of cream per day, which led to the consolidation phase and clearing of most of the lesions; however, with time, the patient could not keep up with her therapy regime. During the last 3 years, the patient complained of persisting severe itching and presented exclusively disseminated nodules (**Figure 1D**). Oral tetracycline in combination with vitamin PP was partially helpful in controlling the disease. The addition of mirtazapine and escitalopram significantly decreased the intensity of pruritus, but did not provide complete remission. The patient has still experienced several alternating periods of remission and relapse.

## Discussion

There are several publications on PN in the literature, mainly single case reports (4, 5, 10, 13). All authors have agreed that the correct diagnosis and treatment of the patients with PN remains a challenge. If the patients present from the beginning of the disease disseminated nodules accompanied by pruritus, they are recognized as having and are treated for prurigo nodularis for a long time. If diagnostic procedures are not performed at that time, the correct diagnosis and therapy are delayed.

Here, we report a large case series on PN, involving 5 patients with many years of follow-up. All of them were women, over 60 years of age (from 62 to 85), and this is consistent with most of the patients described in the literature; however, there are a few articles on PN in children (14, 15).

The patients presented in our case series were divergent in terms of clinical and immunological features as well as long-lasting course and therapy.

Clinically, among our patients, two presented only nodules disseminated on the trunk and extremities throughout their disease course, and two others initially developed classic BP with disseminated blisters and erythematous lesions, which, after years, transformed into exclusively PN (4, 14–16). That was consistent with previously reported cases. Thus, one should be aware that PN may be a secondary presentation of a classic bullous pemphigoid. PN may be generalized or limited to one area of the body (16–18). The remaining patient in our group fulfilled the clinical criteria of MMP at the beginning, presenting oral erosions and scarring conjunctivitis, subsequently developing numerous nodules on the trunk and upper and lower extremities. As far as we know, this is the first description of nodular MMP. This patient for several years presented as classic MMP and then developed disseminated nodular lesions. Since she complained of anxiety problems, she was considered as having prurigo nodularis, but repeated DIF of the skin disclosed IgG and IgA deposits at the BMZ, characteristic of MMP. It is important to emphasize that all our patients reported very severe pruritus for the duration of the disease, even during the clinical remission periods. It is widely accepted that pruritus is a strong trait of BP with an intensity much stronger than psoriasis and comparable with that observed in atopic dermatitis as we proved in our previous study (19). It is accepted that pruritus in BP is associated with elevated IgE serum levels, but its nature in other blistering disorders like MMP, EBA, or epidermolysis bullosa is not clear. On the other hand, itching may be a result of many factors such as dry skin, old age, drugs taken, comorbidities, i.e., type II diabetes mellitus, kidney diseases, and psychological status. All these conditions lead to scratching and releasing of pruritic cytokines with sustained itching. Thus, it is likely that overlapping of these conditions may be responsible for itching and the subsequent development of prurigo nodularis-like lesions.

The etiology of PN is completely unknown, though the disease may be caused by drugs, like nifedipine, etanercept, dipeptidyl-peptidase 4 inhibitor, psoralens, and PUVA (20–24). Interestingly, there are a few reports on PN coexisting with rheumatoid arthritis, lichen planus, psoriasis, or diabetes—diseases that are pruritic (20, 22, 24–30). It is highly likely that chronic, severe itching in these cases led to the damage of BMZ and exposition of its antigens and subsequently to the PN development. In our patients, drugs were excluded as provocative factors; however, some of them had emotional problems of various intensities, which are known to aggravate the itching.

Immunologically, classic BP is well characterized by the development of circulating IgG antibodies against the NC16a domain of BP180 and the presence of IgG and C3 deposits along the BMZ by DIF (1). Among our patients, all had positive DIF and all but one had circulating IgG antibodies labeling the roof of the artificial blister in SS-IIF. Additional studies (ELISA and BIOCHIP) disclosed, in two patients, reaction of circulating IgG antibodies with the NC16a domain of BP180 and/or BP230, like in several cases with PN in the literature. In other words, two of our patients failed to detect any target antigens despite using numerous techniques. This was observed in other papers. It may be associated with low titer of circulating antibodies in atypical forms of pemphigoid, i.e., nodular or localized BP as disclosed by Kawahara et al. (31). It was also shown that patients with PN may have circulating antibodies directed to other BP180 antigens of BMZ, like laminin γ1, which is characteristic of a separate entity: anti-laminin γ1 pemphigoid (32). In the current study, we showed for the first time that nodules may also develop in a course of MMP. The diagnosis in that case was established using not only serological tests but also FOAM-LSCM on the patient’s tissue (33). Therefore, we postulate that PN seems to be not a subtype of bullous pemphigoid, but a symptom of different blistering disorders (anti-laminin γ1 pemphigoid, MMP). That is why it is mandatory to perform detailed tests to establish the proper diagnosis as it determines proper management. Moreover, in case of MMP, it is necessary to periodically examine the patient in terms of progress of skin and mucosal lesions, i.e., development of scarring conjunctivitis or esophagitis, as it was with our patient.

On the basis of the literature, patients with PN were extremely challenging since they were treated using various methods like oral steroids, azathioprine, cyclophosphamide, IVIG, dapsone, minocycline, methotrexate, and others with different results (27, 30, 34–37). According to the European guideline of BP, cream with clobetasol propionate on the entire body is recommended as a first-line therapy since majority of BP patients are elderly with numerous internal disorders (1). Moreover, it is worth mentioning that many patients with pemphigoid have neurological and/or psychiatric disorders (not infrequently associated with pruritus), which undoubtedly affect treatment compliance and may explain such enormous therapeutic difficulties. On the other hand, different medications may trigger BP, mainly diuretics, ACEI, and some antibiotics. In the literature, there are only a few papers on the provocative role of antidepressants in BP (38–42). In general, patients with BP should always be analyzed in terms of drugs they take; however, in a BP patient who experienced extensive pruritus (no response to common antipruritic drugs), antidepressants are worth consideration, because benefits from such therapy may be superior to the risk of BP exacerbation as we observed in presented patients. Four of our 5 patients had depression or anxiety disorder; therefore, in three of them, antidepressants were added to the dermatological treatment leading to the fairly good control of pruritus and skin lesions (see **Table 1**). The patient with nodular MMP (P4) has been treated all the time with dapsone with good control of oral lesions and conjunctivitis. However, dapsone did not control pruritus and nodules at all. In this patient, different combinations of antidepressants were tried, finally attaining success after clomipramine with quetiapine. This particular case suggests that, in some cases, the efforts should be made to find the right medications to control the pruritus.

In terms of course of the disease, in one of our patients (P3), it was relatively mild, similarly to the course of classical BP, i.e., the 7-month therapy led to the complete long-term remission. The remaining four patients presented chronic and recurrent course of the disease, resistant to many therapies. This could have been caused by chronic itching of the skin leading to the scratching and the formation of new nodules. Finally, adding antidepressants allowed for reduction of pruritus and enabled the healing of nodules.

In conclusion, this paper disclosed that:

1. Patients with PN are immunologically heterogeneous; therefore, there is a need for precise analysis of target antigens recognized by circulating antibodies since this determines their management.
2. Patients with PN should be verified in terms of coexisting emotional problems similarly to those with prurigo nodularis.
3. In most of the cases with PN, clobetasol propionate in cream as the first-line treatment should be considered.
4. In case of PN with very severe itching, adding antidepressants to clobetasol propionate might be considered.

## Data Availability Statement

The raw data supporting the conclusions of this article will be made available by the authors, without undue reservation.

## Ethics Statement

The studies involving human participants were reviewed and approved by the Bioethical Committee of the Medical University of Warsaw, Warsaw, Poland. The patients/participants provided their written informed consent to participate in this study.

## Author Contributions

All had full access to all of the data in the study and responsibility for the integrity of the data. Accuracy of the data analysis: KS, AA, CK, and KW. Study concept and design: KW, KS, and CK. Acquisition of data: KS, AA, BJ, EP and KW. Analysis and interpretation of data: KS, AA, KW, BJ, EP and CK. Drafting of the manuscript: KS and KW. Critical revision of the manuscript for important intellectual content: CK and KW. All authors contributed to the article and approved the submitted version.

## Funding

This work was supported by a grant from the Ministry of Science and Higher Education, Poland (no. 2 P05B 065 30) and by a grant from the National Center of Science, Poland (no. N N402 661940).

## Conflict of Interest

The authors declare that the research was conducted in the absence of any commercial or financial relationships that could be construed as a potential conflict of interest.

## Publisher’s Note

All claims expressed in this article are solely those of the authors and do not necessarily represent those of their affiliated organizations, or those of the publisher, the editors and the reviewers. Any product that may be evaluated in this article, or claim that may be made by its manufacturer, is not guaranteed or endorsed by the publisher.

## Abbreviations

ACEI, angiotensin-converting-enzyme inhibitors; BMZ, basement membrane zone; BMZ, basement membrane zone; BP, bullous pemphigoid; CHD, coronary heart disease; COPD, chronic obstructive pulmonary disease, DIF, direct immunofluorescence; EBA, Epidermolysis bullosa acquisita; ELISA, enzyme-linked immunosorbent assay; FOAM-LSCM, fluorescence overlay antigen mapping using laser scanning confocal microscopy; HF, heart failure; HT, hypertension; IIF, indirect immunofluorescence; IIF, indirect immunofluorescence; IVIG, intravenous immune globuli; MMP, mucous membrane pemphigoid, ND, not done; PBC, primary biliary cirrhosis; PN, Pemphigoid nodularis; PUVA, photochemotherapy; SS, salt split SS-IIF, salt split skin substrate; T2DM, type 2 diabetes.

## References

[1] Feliciani C Joly P Jonkman MF Zambruno G Zillikens D Ioannides D. Management of Bullous Pemphigoid: The European Dermatology Forum Consensus in Collaboration With the European Academy of Dermatology and Venereology. Br J Dermatol (2015) 172(4):867–77. doi: 10.1111/bjd.13717 25827742

[2] Montagnon CM Tolkachjov SN Murrell DF Camilleri MJ Lehman JS. Subepithelial Autoimmune Blistering Dermatoses: Clinical Features and Diagnosis. J Am Acad Dermatol (2021) 85(1):1–14. doi: 10.1016/j.jaad.2020.11.076 33684496

[3] Yoshimoto N Ujiie H Hirata Y Izumi K Nishie W Shimizu H. Bullous Pemphigoid Developed in a Patient With Prurigo Nodularis. J Eur Acad Dermatol Venereol (2017) 31(4):e187–e9. doi: 10.1111/jdv.13911 27511319

[4] Vornicescu C Senila SC Cosgarea R Candrea E Pop AD Ungureanu L. Pemphigoid Nodularis - Rare Presentation of Bullous Pemphigoid: A Case Report and Literature Review. Exp Ther Med (2019) 17(2):1132–8. doi: 10.3892/etm.2018.7057 PMC6327548 30679985

[5] Yung CW Soltani K Lorincz AL. Pemphigoid Nodularis. J Am Acad Dermatol (1981) 5(1):54–60. doi: 10.1016/S0190-9622(81)70077-X 7024336

[6] Kwon CD Khanna R Williams KA Kwatra MM Kwatra SG. Diagnostic Workup and Evaluation of Patients With Prurigo Nodularis. Medicines (Basel) (2019) 6(4):1–10. doi: 10.3390/medicines6040097 PMC6963711 31561504

[7] Gammon WR Kowalewski C Chorzelski TP Kumar V Briggaman RA Beutner EH. Direct Immunofluorescence Studies of Sodium Chloride-Separated Skin in the Differential Diagnosis of Bullous Pemphigoid and Epidermolysis Bullosa Acquisita. J Am Acad Dermatol (1990) 22(4):664–70. doi: 10.1016/0190-9622(90)70094-X 2180996

[8] Hashimoto T Tsuruta D Koga H Fukuda S Ohyama B Komai A. Summary of Results of Serological Tests and Diagnoses for 4774 Cases of Various Autoimmune Bullous Diseases Consulted to Kurume University. Br J Dermatol (2016) 175(5):953–65. doi: 10.1111/bjd.14692 27106498

[9] Adaszewska A Kalinska-Bienias A Jagielski P Wozniak K Kowalewski C. The Use of BIOCHIP Mosaics in Diagnostics of Bullous Pemphigoid: Evaluation and Comparison to Conventional Multistep Procedures. J Cutan Pathol (2020) 47(2):121–7. doi: 10.1111/cup.13591 31603994

[10] Al-Salhi W Alharithy R. Pemphigoid Nodularis. J Cutan Med Surg (2015) 19(2):153–5. doi: 10.2310/7750.2014.14018 25775623

[11] Wozniak K Kazama T Kowalewski C. A Practical Technique for Differentiation of Subepidermal Bullous Diseases: Localization of *In Vivo*-Bound IgG by Laser Scanning Confocal Microscopy. Arch Dermatol (2003) 139(8):1007–11. doi: 10.1001/archderm.139.8.1007 12925388

[12] Joly P Roujeau JC Benichou J Picard C Dreno B Delaporte E. A Comparison of Oral and Topical Corticosteroids in Patients With Bullous Pemphigoid. N Engl J Med (2002) 346(5):321–7. doi: 10.1056/NEJMoa011592 11821508

[13] Tani M Murata Y Masaki H. Pemphigoid Nodularis. J Am Acad Dermatol (1989) 21(5 Pt 2):1099–104. doi: 10.1016/S0190-9622(89)70305-4 2681295

[14] Ratnavel RC Shanks AJ Grant JW Norris PG. Juvenile Pemphigoid Nodularis. Br J Dermatol (1994) 130(1):125–6. doi: 10.1111/j.1365-2133.1994.tb06899.x 8305303

[15] Das D Bandyopadhyay D. Juvenile Pemphigoid Nodularis: Report of a Rare Case. Indian Dermatol Online J (2014) 5(2):189–92. doi: 10.4103/2229-5178.131101 PMC4030353 24860760

[16] Tashiro H Arai H Hashimoto T Takezaki S Kawana S. Pemphigoid Nodularis: Two Case Studies and Analysis of Autoantibodies Before and After the Development of Generalized Blistering. J Nippon Med Sch (2005) 72(1):60–5. doi: 10.1272/jnms.72.60 15834209

[17] Zhang W Liu Y Li C. Generalised Nodules in Pemphigoid Nodularis. Lancet (2017) 389(10082):1930. doi: 10.1016/S0140-6736(17)30064-8 28215664

[18] Borradori L Prost C Wolkenstein P Bernard P Baccard M Morel P. Localized Pretibial Pemphigoid and Pemphigoid Nodularis. J Am Acad Dermatol (1992) 27(5 Pt 2):863–7. doi: 10.1016/0190-9622(92)70268-K 1469147

[19] Kalinska-Bienias A Kowalczyk E Jagielski P Lesniewska A Komorowska A Kowalewski C. Clinical Characteristics of Pruritus in Patients With Bullous Pemphigoid: A Preliminary Questionnaire-Based Study. Postepy Dermatol Alergol (2020) 37(6):938–42. doi: 10.5114/ada.2020.102111 PMC7874859 33603613

[20] Mochizuki M Fujine E Tawada C Kanoh H Seishima M. Pemphigoid Nodularis Possibly Induced by Etanercept. J Dermatol (2013) 40(7):578–9. doi: 10.1111/1346-8138.12171 23594393

[21] Powell AM Albert S Gratian MJ Bittencourt R Bhogal BS Black MM. Pemphigoid Nodularis (non-Bullous): A Clinicopathological Study of Five Cases. Br J Dermatol (2002) 147(2):343–9. doi: 10.1046/j.1365-2133.2002.04754.x 12174109

[22] Tahara J Ono S Nomura T Kaku Y Egawa G Dainichi T. A Case of Dipeptidyl-Peptidase 4 Inhibitor-Associated Pemphigoid Nodularis. Int J Dermatol (2021) 60(9):1159–60. doi: 10.1111/ijd.15458 33660842

[23] Ameen M Harman KE Black MM. Pemphigoid Nodularis Associated With Nifedipine. Br J Dermatol (2000) 142(3):575–7. doi: 10.1046/j.1365-2133.2000.03389.x 10777269

[24] Amber KT Korta DZ de Feraudy S Grando SA. Vesiculobullous Eruption in a Patient Receiving Psoralen Ultraviolet A (PUVA) Treatment for Prurigo Nodules: A Case of PUVA-Aggravated Pemphigoid Nodularis. Clin Exp Dermatol (2017) 42(7):833–5. doi: 10.1111/ced.13172 PMC6075654 28597976

[25] Koga H Hamada T Ohyama B Nakama T Yasumoto S Hashimoto T. An Association of Idiopathic Chronic Eosinophilic Pneumonia With Pemphigoid Nodularis: A Rare Variant of Bullous Pemphigoid. Arch Dermatol (2009) 145(11):1339–40. doi: 10.1001/archdermatol.2009.273 19917977

[26] Gao XH Lin J Yang C Ma L Wang G Wang Y. A Case of Kaposi’s Sarcoma Associated With Pemphigoid Nodularis. J Dermatol (2001) 28(7):388–92. doi: 10.1111/j.1346-8138.2001.tb00155.x 11510508

[27] Sakuma-Oyama Y Powell AM Albert S Oyama N Bhogal BS Black MM. Lichen Planus Pemphigoides Evolving Into Pemphigoid Nodularis. Clin Exp Dermatol (2003) 28(6):613–6. doi: 10.1046/j.1365-2230.2003.01401.x 14616828

[28] McGinness JL Bivens MM Greer KE Patterson JW Saulsbury FT. Immune Dysregulation, Polyendocrinopathy, Enteropathy, X-Linked Syndrome (IPEX) Associated With Pemphigoid Nodularis: A Case Report and Review of the Literature. J Am Acad Dermatol (2006) 55(1):143–8. doi: 10.1016/j.jaad.2005.08.047 16781310

[29] Kwong HL Lim SP. Pemphigoid Nodularis Mimicking Nodular Prurigo in an Immune-Suppressed Patient With Rheumatoid Arthritis. Acta Derm Venereol (2015) 95(2):237–8. doi: 10.2340/00015555-1904 24909759

[30] Teraki Y Fukuda T. Pemphigoid Nodularis Associated With Psoriatic Erythroderma: Successful Treatment With Suplatast Tosilate. Br J Dermatol (2008) 158(2):424–6. doi: 10.1111/j.1365-2133.2007.08333.x 18047503

[31] Kawahara Y Matsumura K Hashimoto T Nishikawa T. Immunoblot Analysis of Autoantigens in Localized Pemphigoid and Pemphigoid Nodularis. Acta Derm Venereol (1997) 77(3):187–90. doi: 10.2340/0001555577187190 9188867

[32] Tomura Y Noto M Ishii N Hashimoto T Manabe M Osada SI. Nodular Formation in Anti-Laminin Gamma1 Pemphigoid. J Dermatol (2020) 47(3):e80–e2. doi: 10.1111/1346-8138.15227 31916269

[33] Wozniak K Jakubowska B Kalinska-Bienias A Hashimoto T Ishii N Kowalewski C. Diagnosis of Autoimmune Subepidermal Bullous Diseases With Mucous Membrane Involvement Based on Laser-Scanning Confocal Microscopy. Eur J Dermatol (2020) 30(5):516–23. doi: 10.1684/ejd.2020.3765 32972911

[34] Hattori M Shimizu A Ishikawa O. Development of Pemphigoid Nodularis After Remission of Bullous Lesions. Clin Exp Dermatol (2019) 44(2):e1–2. doi: 10.1111/ced.13771 30242789

[35] Vila-Payeras A Dominguez-Mahamud C Terrasa-Sagrista F Vila-Mas A Parera-Amer E Nadal-Llado C. Pemphigoid Nodularis: An Infrequent Variant Responding to Rituximab. Australas J Dermatol (2020) 61(4):e438–e9. doi: 10.1111/ajd.13331 32408380

[36] Shintani T Ohata C Koga H Ohyama B Hamada T Nakama T. Combination Therapy of Fexofenadine and Montelukast is Effective in Prurigo Nodularis and Pemphigoid Nodularis. Dermatol Ther (2014) 27(3):135–9. doi: 10.1111/dth.12094 24102897

[37] Gach JE Wilson NJ Wojnarowska F Ilchyshyn A. Sulfamethoxypyridazine-Responsive Pemphigoid Nodularis: A Report of Two Cases. J Am Acad Dermatol (2005) 53(2 Suppl 1):S101–4. doi: 10.1016/j.jaad.2004.07.062 16021154

[38] Mehravaran M Gyulai R Husz S Dobozy A. Drug-Induced Erythema Multiforme-Like Bullous Pemphigoid. Acta Derm Venereol (1999) 79(3):233. doi: 10.1080/000155599750011066 10384926

[39] Rault S Grosieux-Dauger C Verraes S Bernardeau K Durlach A Bernard P. Bullous Pemphigoid Induced by Fluoxetine. Br J Dermatol (1999) 141(4):755–6. doi: 10.1046/j.1365-2133.1999.03130.x 10583137

[40] Caccavale S Mea EE La Montagna M. Bullous Pemphigoid Induced by Escitalopram in a Patient With Depression. G Ital Dermatol Venereol (2016) 151(1):122–3. 26924032

[41] Naramala S Dalal H Adapa S Patel P Konala VM. Hydrochlorothiazide vs Venlafaxine: Drug-Induced Bullous Pemphigoid. Cureus (2019) 11(6):e4999. doi: 10.7759/cureus.4999 31497430 PMC6713256

[42] Lloyd-Lavery A Chi CC Wojnarowska F Taghipour K. The Associations Between Bullous Pemphigoid and Drug Use: A UK Case-Control Study. JAMA Dermatol (2013) 149(1):58–62. doi: 10.1001/2013.jamadermatol.376 23324757

